# High incidence of stillbirths in a free farrowing system linked to uterotonic misuse and improper farrowing management: a case report

**DOI:** 10.1186/s40813-025-00466-1

**Published:** 2025-11-20

**Authors:** Alexander Grahofer, Heiko Nathues, Jens Becker

**Affiliations:** 1 Clinic for Swine, Department of Clinical Veterinary Science, Vetsuisse Faculty, University of Bern, Bremgartenstrasse 109a, Bern, 3012 Switzerland; 2 Clinic for Ruminants, Department of Clinical Veterinary Science, Vetsuisse Faculty, University of Bern, Bern, 3012 Switzerland

**Keywords:** Farrowing management, Farrowing pen, Stillborn, Animal welfare, Hormone, Parturition

## Abstract

**Background:**

An aceptable target range for stillbirths per litter varies between 5–7% of total born piglets in sows. Several major risk factors have been identified that contribute to the increased incidence of stillbirths in piglet-producing herds, including the use of uterotonic agents. Oxytocin and carbetocin are commonly administered to manage the farrowing process. Oxytocin is a short-acting, natural hormone that induces rapid uterine contractions, while carbetocin is a long-acting synthetic analog designed for prolonged stimulation. Both agents can affect stillbirth rates depending on timing of application, dosage, and the sow’s physiological condition, emphasizing the need for cautious and informed use.

**Case presentation:**

A Swiss piglet-producing herd suffered from an increased stillbirth rate of 8.7%. A herd examination was conducted to reveal the general health status of the herd. The birth process of eight sows, resulting in a total of 129 piglets, was analysed for birth management, total duration of birth, and duration of piglet expulsion. Each piglet was scored for meconium staining and vitality. In addition, material from stillborn and weak-born piglets was subjected to further examinations. The general physical examination of the sows before farrowing revealed no abnormalities. At different time points during the farrowing process, all sows routinely received an intramuscular treatment of 35 µg carbetocin once by the animal caretaker, which caused a prolonged piglet-to-piglet interval directly after application, loss of colostrum and an increased number of weak and stillborn piglets. Histological examination of heart samples from five stillborn piglets revealed no signs of myocarditis or other abnormalities. Moreover, qPCR for porcine circovirus type 2 on the heart samples was negative. Serology on pre-colostral serum samples of one litter with a mummified piglet were negative for antibodies towards porcine parvovirus. Porcine reproductive and respiratory syndrome virus was excluded by PCR examination of the serum of ten weak-born piglets. After stopping the routine treatment with carbetocin and improving the birth management, both recommended by the examination team, the level of stillbirths decreased to 4.6%.

**Conclusion:**

Herd problems with stillbirths require a comprehensive herd investigation including monitoring the birth management and ruling out potential pathogens. In this case, the routinely administration of carbetocin during parturition led to severe undesirable side effects. A good monitoring during the farrowing process combined with appropriate measures and the omission of prophylactic carbetocin administration enhanced the birth process and thereby piglets’ survival.

## Background

**Fig. 1:**
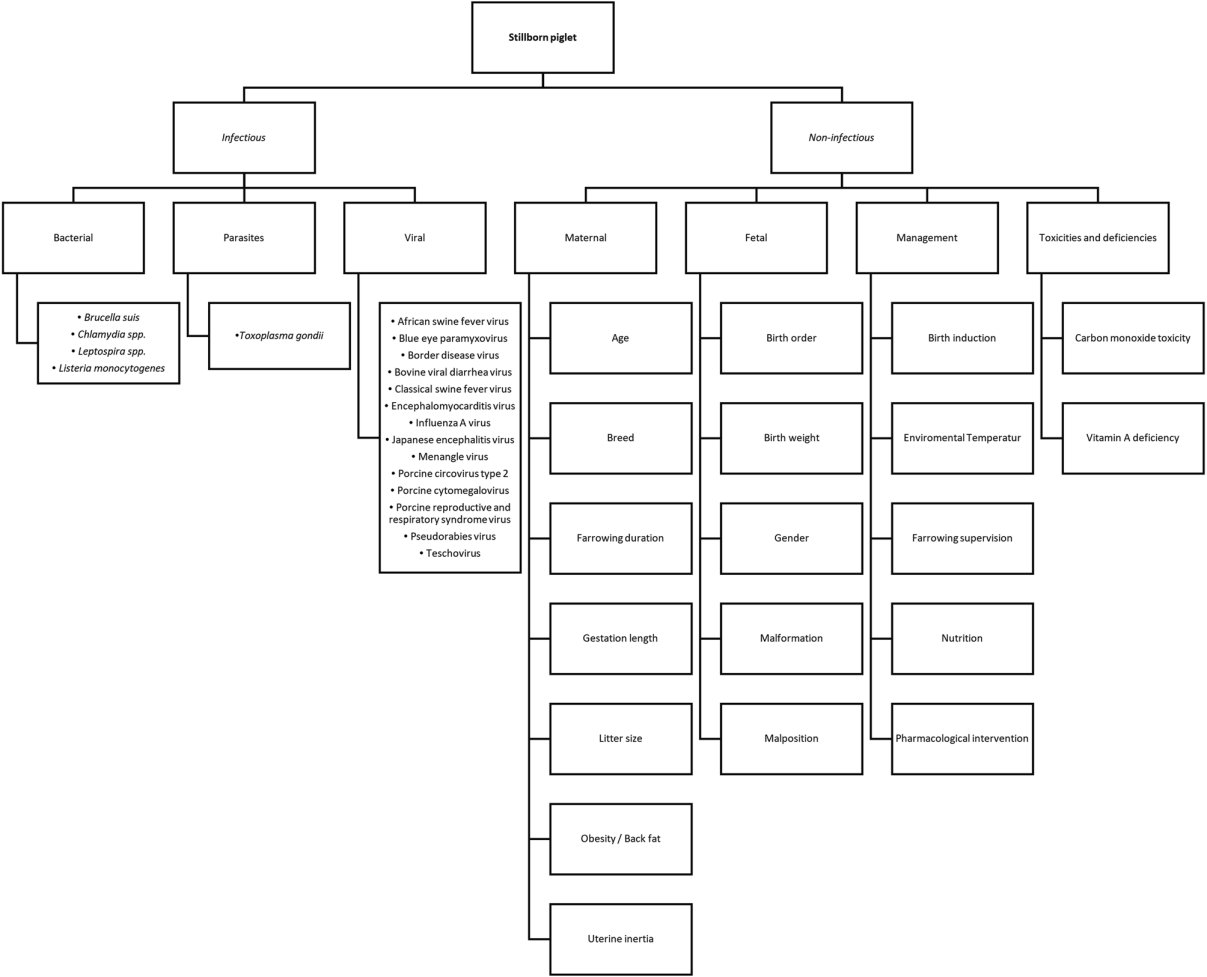
Overview of most relevant causes for stillborn piglets in sows

While a stillborn rate of 5–7% of is common in hyper-prolific sows, values above this target require management adaptions following diagnostic investigations [1–3]. In addition to economic loss, increased numbers of stillborn piglets are considered a welfare concern [4]. Risk factors for stillbirth in piglets are numerous and include non-infectious as well as infectious causes (Fig. 1) [1, 5–9].

A significant risk factor for stillbirths in swine parturition management arises from the inappropriate administration of pharmacological agents, particularly the excessive use of uterotonics [10–12]. Uterotonic agents have been repeatedly reported to negatively influence the dynamics of the foetal expulsion and are correlated with an increased incidence of adverse neonatal outcomes, including stillbirth, umbilical cord pathologies, and meconium staining [13–15].

During parturition, oxytocin plays a crucial role in regulating the powerful, rhythmic contractions of the myometrium [16–18]. This process operates through a positive feedback loop involving the posterior lobe of the pituitary gland. When oxytocin is released, it stimulates strong myometrial contractions, which increase the pressure exerted by the fetus on the cervix. Increased cervical stimulation triggers the release of more oxytocin from the pituitary gland which further amplifies the contractions [17]. The oxytocin profile during this stage is complex and pulsatile, ensuring that uterine activity is sustained and effective for the progression of labor [17].

In the farrowing management, oxytocin and carbetocin are uterotonic agents often used to promote uterine contractions [19–31]. Oxytocin, either alone or in combination with other injectable medications, is routinely administered on approximately 9% of swine farms in the United States to support normal parturition in sows. [32]. While oxytocin is a naturally occurring hormone, carbetocin is a long-acting synthetic analogue specifically designed to mimic and extend oxytocin effects [33, 34]. Structurally, carbetocin is a modified octapeptide-one-deamino-one-carba-2-tyrosine (O-methyl)-oxytocin with agonist activity at oxytocin receptors [35, 36]. Both agents exert their effects by binding to oxytocin receptors located on the smooth muscle of the uterus, promoting rhythmic contractions, enhancing the strength and frequency of existing contractions, and increasing overall uterine tone [36]. Despite these similarities, carbetocin differs significantly in its pharmacokinetic profile. Although carbetocin demonstrated about 50% less myometrial contractile efficacy compared to oxytocin in vivo [36], intramuscular administration of carbetocin in humans produced rapid tetanic contractions of the uterus within two minutes, lasting approximately 11 minutes, followed by sustained rhythmic contractions for up to two hours [35]. This is in contrast with the effect of oxytocin, which has a shorter duration of action and half-life of 1–5 minutes [37]. An overview of the scientifically reported use of oxytocin and carbetocin in sows throughout the peripartal period is provided in Table 1.

**Table 1:** Overview of the scientifically reported administration of oxytocin and carbetocin in sows during the peripartal period

Usage	Oxytocin	Carbetocin
Birth induction with PGF₂α	• 10 IU Decaluwé et al., 2014 [38]	• 0.6 µg/kg Boonraungrod et al., 2018 [19] • 35 µg Wehrend et al., 2005 [20]; Zaremba et al., 2019 [21] • 70 µg Zaremba et al., 2019 [21]
Stimulation of myometrial contractions for fetal expulsion	• 5–10 IU Peltoniemi et al., 2019 [10]; Björkman & Grahofer, 2020 [11] • 10 IU Ward et al., 2019 [22] • 20 IU Hühn et al., 2004 [23]; Jiarpinitnum et al. 2019 [24]; Bill et al., 2021 [25]; Jahn et al., 2022 [26] • > 20 IU more side effects than benefits [12]	• 0.4 µg/kg Juthamanee et al., 2024 [27] • 0.5 µg/kg Vongsariyavanich et al., 2021 [28] • 0.6 µg/kg Jiarpinitnum et al., 2019 [24] • 70 µg Hühn et al., 2004 [23]; Ward et al., 2019 [22] 140 µg Hühn et al., 2004 [23]
Stimulation of myometrial contractions for placental expulsion	• 10 IU Peltoniemi et al., 2016 [29]	–
Support of uterine involution	• 5 IU Björkman et al., 2018 [30]	–
Stimulation of milk letdown	• 5–10 IU Cort et al., 1982 [31]; Martineau, 2005 [39]	–

IU = International Unit

Limited information is available regarding the usage of uterotonics parturition in free farrowing sows [13, 25]. In these studies, the administration of oxytocin after the first or fourth piglet had no significant effect on farrowing duration [13, 25]. A significant increase in intrapartum deaths, particularly cases involving ruptured umbilical cords and severe meconium staining, was observed in oxytocin-treated sows [13]. Hence the routine usage of uterotonics in free farrowing sows, may be associated with more side effects than in crated sows [12, 13, 25, 40].

This case report documents an elevated incidence of stillbirths in a Swiss free farrowing system, potentially associated with the routine administration of a single carbetocin injection at varying stages of parturition, in conjunction with suboptimal farrowing management.

## Case presentation

A piglet producing herd in Switzerland had been experiencing an increased stillbirth rate for several months. The farm consisted of 112 sows and one boar and was run by the owner and two employees. The boar had been replaced two years prior to the visit. Every six weeks, five gilts of approximately 200 days of age were introduced directly into the herd from the same breeder without performing quarantine upon arrival, resulting in a annual replacement rate of 40%. The gilts were first inseminated between 221 and 242 days of age with an average body weight of 140–150 kg. The farm used a three-week batch farrowing system (16 sows per group) with an average lactation period of 26 days. Overall, 90% of the sows were artificially inseminated intracervically. In the other sows, natural mating was implemented. After the last insemination, the sows were moved from the insemination crates into the group housing system. The gestation unit consisted of an indoor area, which was structured into several berths with deep straw-beeding and an outdoor paddock with concrete flooring, an automatic feeding system and a hay rack. During summer, awning and water sprinklers were available at outdoor area.

The sows were fed a commercial liquid diet. After weaning, flushing with 250 g dextrose per day and animal was implemented until insemination. During the gestation period, sows were fed gestation feed twice daily. The sows were moved into the free farrowing pen 7–10 days before the calculated farrowing date. Until farrowing, twice daily, sows were fed a liquid diet consisting of 50% gestation feed and 50% lactation feed. In this period, sows also received 250 g of dextrose *per os* once daily. After farrowing, sows were fed commercial lactation feed thrice daily. Public spring water was available for the sows ad libitum through a nipple drinker system. The following vaccination regime was implemented on the farm. Prior to purchase, gilts received a basic immunisation against porcine circovirus type 2 and against erysipelas and parvovirosis by intramuscular route. During the gestation period, gilts received basic immunization against *Escherichia coli* and *Clostridium perfringens* intramuscularly six weeks and three weeks prior to farrowing. Sows were administered a booster immunization against erysipelas and parvovirosis by intramuscular route on approximatively day 13 of lactation.

Prior to conducting the herd examination, performance data of the previous year were analyzed (Table 2).

**Table 2:** Performance data of sows in a Swiss free farrowing system of the year prior to examination due to an increased rate of stillborn piglets (before intervention) and performance characteristics six months after implementing measures (after intervention)

Performance data	Before intervention	After intervention	Reference*
Live born piglets/sow/litter (n)	13.2	14.3	≥12
Stillborn piglets/sow/litter (%)	8.7	4.6	< 7
Litters/sow/year (n)	2.4	2.4	≥2.4
Weaned piglets/sow/year (n)	23.1	26.5	≥25
Return to estrus (%)	5.7	5.8	≤12
Farrowing rate (%)	88.7	89.1	≥90
Replacement rate (%)	40	40	35–40
Pre-weaning mortality rate (%)	25.2	12.8	< 13

* grosse Beilage et al. 2013, (14)

**Fig. 2:**
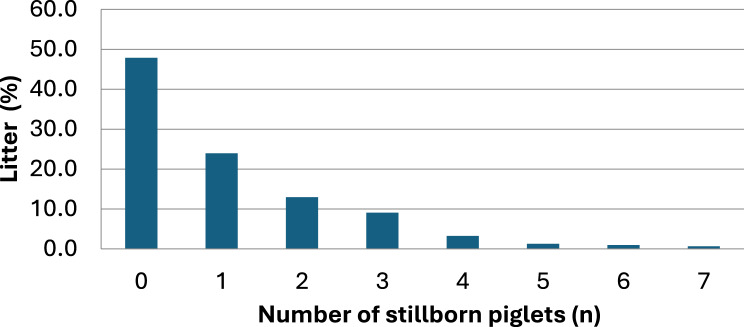
Percentage of litters grouped by the number of stillborn piglets of the last 12 months before intervention

**Fig. 3:**
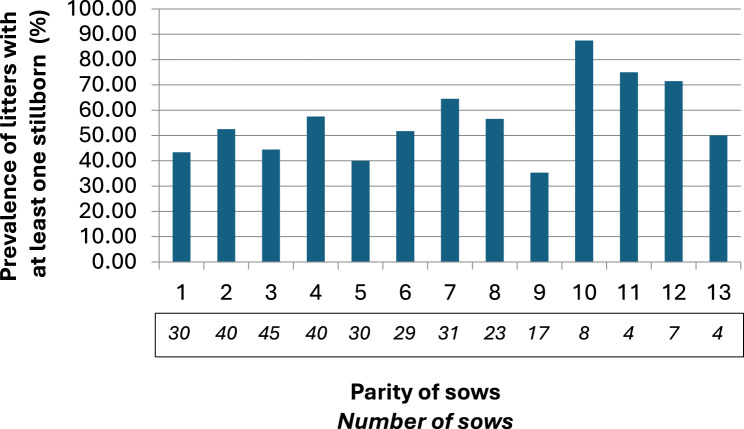
Prevalence of litters with at least one stillborn piglets per parity in a Swiss farm with free farrowing, experiencing high incidence of stillborn before intervention

A detailed analysis of the litters was conducted, revealing that in more than 50% of the litters at least one stillborn piglet was present (Fig. 2). No association of parity with the number of stillborn piglets was observed using Kruskal-Wallis One-Way ANOVA (*p* = 0.36) (Fig. 3). Similarly, neither an association of farrowing batch with the number of stillborn piglets (*p* = 0.18) nor a seasonal association (*p* = 0.10) were observed.

An increased preweaning mortality was observed, with the highest losses occurring during the first five days post-partum. According to the farm’s recording system, the primary causes of death were crushing and starvation.

After analysis of the performance data, a herd examination was conducted to reveal the general health status of the herd. Clinical examination of lactating sows revealed a Body Condition Score (BCS) of 3–4/5, and physiological mammary complex development. Litters of the sows were heterogeneous and 10% of the piglets showed wasting. Clinical examination of sows in insemination and gestation units revealed good general health conditions and an adequate BCS (insemination unit: 2.5–3/5; gestation unit: 3–4/5). Moreover, the management procedures and housing conditions of the farm were evaluated. The water supply complied with legal standards, and the air quality was in line with established recommendations.

For further diagnostic investigation the birth management was evaluated in the following farrowing batch.

Sows in the farrowing unit received straw (up to 1 kg) once daily to enhance nest building behaviour. The ambient temperature at the farrowing unit was 19.1 °C, whereas the piglet nest temperature varied between 33 and 36 °C. No standard operating procedure for the farrowing monitoring had been implemented on the farm.

The health status and the farrowing process of eight sows were evaluated by the principal investigator. The birth monitoring and management were analysed regarding type and frequency of actions and data collection. Each expelled piglet was classified as live born, stillborn or mummified. Moreover, all piglets were scored for meconium staining [26] and vitality [41]. After the farrowing monitoring, litter vitality was assessed using the scoring system from Schodl et al., 2019 [42].

The median parity of the sows was 4.5 (min:2; max:8) and the BCS was 3.5 (min:3; max:4). The sows showed a good general health condition. During the diagnostic investigation, a worker observed sows in labor at intervals of 30 to 90 minutes.

Sows were routinely administered 35 μg of carbetocin (LongActon®, Vetoquinol, Bern, Switzerland) intramuscularly using a disposable needle (1.6x38mm) at different time points during parturition (between the expulsion of the first piglet and the last born piglet). No specific pattern could be observed, since administration of carbetocin was decided by a worker and not based on a standard operating procedure. No other management procedures such as documentation, piglet care, or obstetrical intervention were implemented on the farm.

**Fig. 4:**
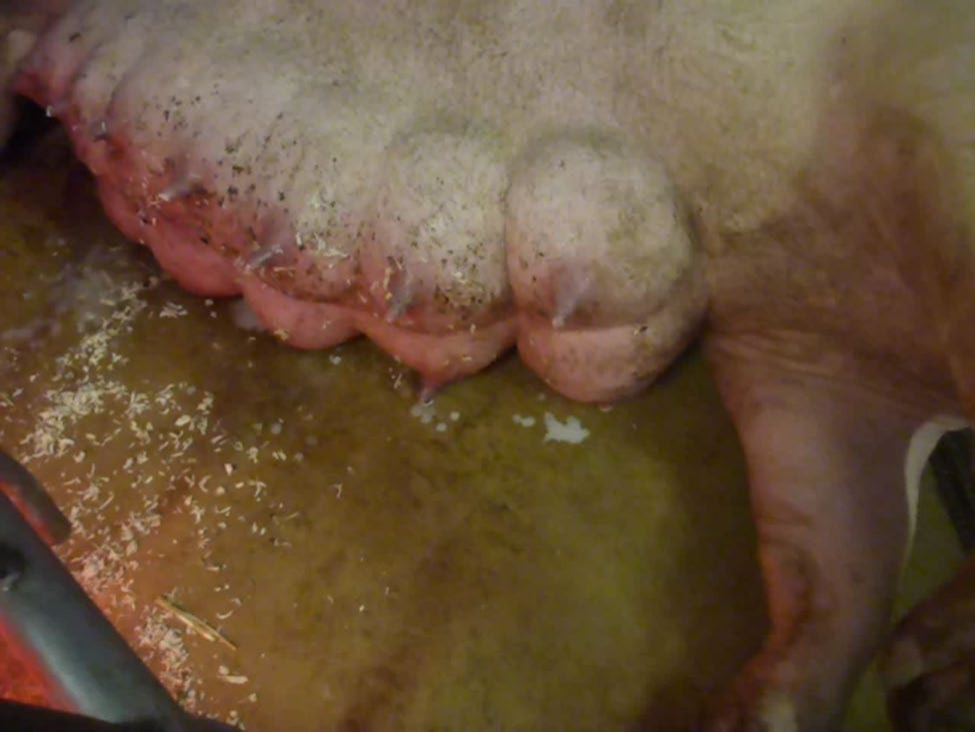
Colostrum leakage in a sow after application of carbetocin on a Swiss farm experiencing high incidence of stillborn piglets

Carbetocin was administered to five sows after the birth of the first piglet, to two sows after the third piglet, and to another sow after the fourth piglet. All sows showed painful abdominal contraction with trembling and pulling the back leg forward or in towards the body. In addition, after administration of carbetocin, colostrum leakage of considerable amounts were observed in six sows (Fig. 4).

Besides the changes in the sow behaviour, the administration of carbetocin also impacted on the farrowing and piglet parameters. The median piglet-to-piglet interval before administration of carbetocin (*n* = 7 piglets), was 19 min on average (min: 1 min; max: 30 min), followed by 55.5 min (min: 20 min; max: 159 min) directly after administration of carbetocin (*n* = 8 piglets). Afterwards, the median decreased to 9 min (min: 0 min; max: 154 min) for all following piglets (*n* = 106 piglets).

After application of carbetocin, five of the subsequently born piglets showed a meconium score of 2 and three had a meconium score of 1. The following percentages of the meconium scoring of the piglets born after administration of carbetocin were observed (*n* = 106 piglets): meconium score (MS) 0: 10.7%; MS 1: 31.0% and MS 2: 58.3%. Vitality scores following administration of carbetocin were: one piglet (12.5%) score 0; four piglets (50%) score 1, and three piglets (37.5%) were scored with score 2. None of the piglets had a physiological score of 3.

Litter vitality scoring revealed three litters with score 1; two litters with score 2 and three litters with score 3. In four pathological births (the first placenta part being expelled prior to birth of the last piglet), the interval between administration of carbetocin to the first part of the placenta part was 70 min, 195 min, 223 min, and 240 min.

In order to exclude infectious causes for stillborn piglets and weak-born piglets, further diagnostic measures were carried out. A total of ten blood samples were taken from the *Vena cava cranialis* of weak-born piglets originating from five litters. Furthermore, 5 stillborn piglets originating from three litters were necropsied on farm, and two heart samples were collected per piglet. Of those, one was immediately stored in formalin for further histological examination, whereas the other part was placed in a labeled container for PCR testing.

Serum samples were tested for Porcine Reproductive and Respiratory Syndrome Virus (PRRSV) using an in-house real-time RT-PCR, and for PRRS antibodies using the commercial IDEXX PRRS X3 Ab test (IDEXX, Westbrook, USA), with both assays yielding negative results for antigen and antibodies. Furthermore, native heart samples were tested for Porcine Cirvovirus type 2 (PCV2) using a quantitative in-house PCR, revealing negative results. The other heart samples underwent histological evaluation, which demonstrated an absence of pathological features consistent with myocarditis.

The evaluation of farrowing management and additional diagnostics, revealed that improper farrowing management, including the misuse of carbetocin during the farrowing process, contributed to the elevated stillbirth rate. Therefore, it was recommended to implement a standard operating procedure for the farrowing management. A continuous farrowing monitoring every 30 minutes including documentation of the farrowing process and proper neonatal care (drying of piglets, approaching piglets to teats, split suckling) had to be implemented. In addition, the farmer was advised to discontinue routine intramuscular treatment with 35 µg carbetocin at any time. In case of dystocia (piglet-to-piglet interval ≥60 min) manual obstetrical intervention should be implemented. For this reason, manual palpation of the birth canal should be carried out following proper cleaning of the vulva with iodine solution and using long gloves with lubricant. In case a fetus is present in the vaginal or cervical canal, then the fetus should be extracted and checking for additional fetuses must be performed. After extraction of fetuses or in case dystocia is present without presence of fetuses, 5–10 IU of oxytocin were to be injected intramuscularly [10, 11].

After six months of initial examination and implementation of the farrowing management, a follow-up was conducted. The performance characteristics on herd and sow level increased (Table 2). Both the stillbirth rate and piglet mortality decreased. The cessation of carbetocin administration was verified through review of the treatment records and confirmed by the herd attending veterinarian. The changes in farrowing management were evaluated by reviewing sow charts documenting the farrowing process, supplemented by detailed explanations provided by the farm owner and staff.

## Discussion and conclusions

The present case report describes a high incidence of stillbirths in a free farrowing farm in Switzerland. A thorough anamnesis, detailed analysis of reproductive data, and subsequent herd examination including an evaluation of farrowing management and ruling out infectious causes, raise concerns about a potential association between carbetocin administration and suboptimal farrowing management and increased stillbirths in piglets, warranting further investigation.

Several studies [14, 22, 27, 28, 36] indicate that factors such as the timing and dosage of uterotonic agents can negatively influence farrowing outcomes and piglet viability. However, the findings of the present case report do not provide sufficient evidence to support a generalized conclusion that extensive use of uterotonics is harmful in all scenarios. In crated sows, routine administration of uterotonic agents has been associated with favorable effects on the farrowing process and related parameters [40]. In contrast, research on their use during parturition in free farrowing systems remains limited [13, 25]. Available studies have shown that administration of oxytocin after delivery of the first or fourth piglet does not significantly alter total farrowing duration [13, 25]. Nevertheless, oxytocin-treated sows exhibited a notable increase in intrapartum piglet deaths, particularly those involving ruptured umbilical cords and severe meconium staining [13]. Taken together, the findings of previous studies, along with the data from this case report, suggest that routine administration of oxytocin or its analogues may not be advisable for free farrowing sows due to the potential adverse outcomes. One plausible mechanism for these effects is the markedly higher endogenous oxytocin concentrations reported in free farrowing sows during the expressed nestbuilding behaviour and the post-expulsion phase of parturition compared with crated sows [43–45]. Another hypothesis is that pig breeds raised in Switzerland exhibit particularly high levels of oxytocin receptors compared with breeds used in crated systems. Evidence from dogs shows that both genetic variation in the oxytocin receptor gene and environmental enrichment can increase oxytocin receptor expression and sensitivity [46]. Therefore, additional exogenous uterotonic agents in this context may induce excessively strong or prolonged uterine contractions, leading to transient umbilical cord compression, impaired fetal oxygenation, and ultimately increased piglet mortality as well as colostrum leakage, paralleling the pathophysiological effects observed with oxytocin overdosing in confined sows [47]. It can be hypothesized that these side effects may be even more pronounced with carbetocin in free farrowing sows, as intramuscular administration induces rapid tetanic contractions within two minutes, lasting for approximately 11 minutes [35]. These effects may have been reflected in the clinical signs observed in all sows, namely painful abdominal contractions accompanied by trembling and pulling the back leg forward or inward toward the body. Furthermore, the prolonged piglet-to-piglet interval, high meconium scores and low piglet and litter vitality may be explained by the tetanic contractions of the uterus. However, no studies to date have specifically investigated the effects of carbetocin in free farrowing systems, underscoring the need for further research to confirm the safety and efficacy of the drug in this context.

The increased stillbirth rate may have been attributed not only to the misuse of carbetocin but also to inadequate farrowing management. An adequate farrowing management requires systematic and frequent monitoring of the sow during parturition to enable timely identification of complications and optimal outcomes for both the sow and piglets. Farm personnel should assess each sow at 30–60 minute intervals throughout the farrowing process, evaluating both the progress of parturition and the condition of the newborn piglets. If the piglet-to-piglet interval exceeds 60 minutes, obstetric intervention should be initiated [10, 11]. The first step involves manual examination of the birth canal and assisted delivery of piglets that can be palpated within the birth canal. If no piglet is present in the birth canal during the examination, sows should receive an intramuscular injection of 5–10 IU oxytocin to stimulate uterine contractions and facilitate further expulsion of fetuses and placental tissues. Consequently, the use of uterotonic agents such as oxytocin or carbetocin is indicated only when no piglet is present in the birth canal upon manual examination after a prolonged interval exceeding 60 minutes following the delivery of the previous piglet. Additionally, newborn piglets should initially be placed under a heat lamp to maintain thermal homeostasis and subsequently encouraged to initiate suckling at the sow’s mammary glands.

Although no additional investigation into the increased preweaning mortality rate was performed, a significant reduction after the recommended and implemented measures were observed on the farm. It is well established that the farrowing process directly influences piglet survival and performance [48, 49]. Prolonged farrowing, due to untreated uterine inertia or large litter size can lead to compromised umbilical blood flow. During uterine contractions, blood vessels supplying the placenta are transiently compressed, which naturally reduces blood flow and oxygen delivery to the fetuses [48, 49]. However, when farrowing is prolonged or contractions become excessively intense or frequent, as may occur with uterotonic administration, as described in this case, the cumulative reduction in placental perfusion can result in significant intrauterine hypoxia. This oxygen deprivation impairs fetal metabolism, increasing the likelihood of weak piglets and stillbirths, as well as reducing overall viability and postnatal survival [48, 49]. Furthermore, affected piglets often have a reduced colostrum intake, which has impact on the energy and immunity level, and therefore are at a higher risk of being crushed due to weakness [49]. Therefore, it can be speculated that the improvements in the farrowing process not only reduce stillbirth rates but also contribute to a decrease in preweaning mortality.

In conclusion, the increased occurrence of intrapartum stillbirths, particularly associated with uterotonic administration during parturition and inadequate farrowing management, represents a notable welfare concern due to the risk of piglet suffocation during delivery. Adjustments to farrowing management practices, along with the discontinuation of carbetocin use, appeared to contribute to a reduction in stillbirth incidence and piglet mortality. These observations suggest that optimizing both management practices and pharmacological interventions in cases of elevated stillbirth rates may enhance neonatal piglet survival, as well as animal health, and thus, overall animal welfare.

## Data Availability

The datasets analysed during the case report are available from the corresponding author on reasonable request.
